# Supplementary material for: Late Permian wood-borings reveal an intricate network of ecological relationships
Source: Nat Commun. 2017 Sep 15;8:556. doi: 10.1038/s41467-017-00696-0 (PMC5601472; doi:10.1038/s41467-017-00696-0)
Supplement: Supplementary file 1 — Supplementary Information [file 41467_2017_696_MOESM1_ESM.pdf]

Description of Supplementary Files

File Name: Supplementary Information
Description: Supplementary Figures, Supplementary Notes and Supplementary References

File Name: Peer Review File

Supplementary Note 1 | A Brief History of Beetle-Borings in Wood

Extant arthropods, particularly insects, often feed in roots, twigs, stems or trunks of woody plants, where they consume bark, phloem, sapwood or heartwood. The feeding behaviour of wood-borers typically involves tunnels through the periderm (bark) or secondary xylem (wood) where the intervening zone (cambium) is generally the targeted tissue for consumption[1,2]. The wood-boring habit, including the detritivorous feeding of oribatid mites and the herbivory of cambium engravers, consists prominently of the activities of larvae and adults from several major and many minor lineages of modern Coleoptera[1]. The principal lineages of wood-boring Coleoptera are jewel beetles (Buprestidae), auger beetles (Bostrychidae), deathwatch beetles (Ptinidae, Anobiinae), ship-timber beetles (Lymexylidae), longhorn beetles (Cerambycidae) and three distinctive subgroups of Curculionidae (weevils): Cossoninae (cossonine weevils), Scolytinae (bark beetles) and Platypodinae (ambrosia beetles)[1,3]. The wood-boring habit also has appeared independently in four basal lineages of symphytan Hymenoptera[4], and sporadically throughout the Lepidoptera, such as many Hepialidae (ghost moths), Cossidae (carpenter worms) and some Noctuidae (owlet moths)[3]. But it is the Coleoptera that has contributed overwhelmingly the greatest diversity of lineages in time and space to the wood borer functional feeding group.

Wood-boring has a long geological history that is closely linked to the fossil record of carbonate- and silica permineralisation of woods. Almost all previous Palaeozoic fossil records of wood-boring have been attributed principally to detritivorous oribatid mites[5,6]. The miniscule tunnels of oribatid mites likely represent the oldest form of wood boring, and have a record of indirect evidence consisting of stereotypical tunnels and their contained coprolite clusters that begins during the Late Devonian[5]. These earliest borings have been reported in progymnosperm tree axes[7,8], and extend to the Permian[9–11], forming an important plant–arthropod interaction of the late Palaeozoic[6,12–15]. After the Devonian, wound response tissue produced by host plants to arthropod borings has been documented in permineralised early Carboniferous

seed-fern stems, which exhibit 3–4 parenchymatous cell layers that are generally flattened and formed the walls of tunnels embedded in phloem tissue[16]. By contrast, borings with considerably larger diameters are absent during the Devonian and early Carboniferous Period, and are encountered rarely during the late Carboniferous Period, becoming somewhat more abundant during the Permian Period. These earliest of the larger-diameter Palaeozoic borings occur on a variety of plant hosts and invariably beetles are the attributed fabricators.

Limited evidence for wood-boring in ancestral beetles may extend to enigmatic, late Carboniferous woods with tunnel diameters too large to be affiliated with oribatid mites[17], and located within the phloem of cordaitalean woods[18,19]. By contrast, the earliest definitive beetle body fossils are early Permian in age[20–22], although an earlier, mid–late Carboniferous occurrence[23], may indicate a previous existence for a stem-group lineage of Coleoptera that created the cordaitalean tunnel networks. More reliably identified beetle borings have been assessed for conifer-like wood from the middle Permian of Tikhie Gory, Russia[24], and late Permian glossopterid tree axis and root woods from Antarctica[25,26]. These data indicate that wood-borer insects initially colonised progymnosperm hosts during the Late Devonian, and subsequently, during the late Carboniferous and Permian, colonised cordaitaleans and conifer-like hosts[27]. With the additional exception of glossopterids, there is no evidence that wood-borer insects ever colonised other late Palaeozoic, arborescent seed plants with wood or other indurated tissues, such as peltasperms, ginkgophytes or cycads.

Beetle activity at the bark–wood interface of conifers has been recognised in some Middle and especially Late Triassic woods from several localities[28,29]. Wood-borings, attributed to beetles, have been recorded in gymnosperm woods, such as *Araucaria*-like wood from the Middle Triassic of Germany[28], and *Agathoxylon* and other conifer wood from the Late Triassic of Arizona, USA[30–32]. However, borings earlier than mid-Jurassic cannot be unambiguously attributed to any, known, living lineage of beetles or other wood-boring insects. The absence of modern affiliations is acknowledged, even though

some ichnogeneric attributions, such as *Paleobuprestis* and *Paleoscolytus*[30] for bored Chinle woods in Arizona may suggest otherwise.

Some of the borings preserved in Late Triassic silicified tree axes from Arizona have been described controversially as termite, bee or wasp nests[33–35]. However, based on a reanalysis of these studies, the putative borings were reinterpreted as probable beetle borings[36]. Although these Late Triassic borings resemble those of anobiid beetles, a group of small beetles that today feed on fungi in wood[37], no living beetle borings closely match the Late Triassic borings. It is possible that another lineage of modern beetles constructed the borings, or alternatively, the borings may have been made by an extinct beetle group[36], or another group of wood-boring insects. Notably, a possible beetle larva has been described from a petrified conifer wood in the Late Triassic Chinle Formation of Arizona[31], as has response tissue from borings[32]. However, the preservation of the body fossil is poor, and the associated tunnel networks lack sclerotised elements with demonstrable segmentation, beetle eggs, fungi and coprolites found in our late Permian material that would result in a more secure determination.

The position, geometry, extent and sequence of borings within late Permian host trees often reflect the consumption strategy of the target tissue by a particular wood-boring species[38,39]. We report that the adult beetles enter through bark and then initially construct a mother tunnel in cambial tissue for consumption and use as an oviposition site[40]. After hatching from oviposited eggs, larvae initially consume cambium and subsequently enter the underlying wood that eventually encloses their tunnels[41]. During the terminal phase of tunneling, the larvae re-emerge from wood, briefly enter the cambium and after pupation the adult exits through the bark[42]. The closure at the terminal phase of this sequence along the cambial and bark tissues from reaction-wood enrollment, destroys evidence for the terminal pupal and exit phases. Nevertheless, this sequence of (i) entry of the adult beetle through bark and into cambium, (ii) hatching of larvae and their tunneling through cambium, (iii) tunneling through wood, (iv) a brief traverse across cambium, and (v) presumed boring through bark as they exit their host

plant represents a formidable dietary challenge during their life cycle. Consumption of these very different tissues would require shifts in the gut microbiome of the larva to include processing of nutrient-rich meristematic tissues, whose food value are only accessible through chitinolytic enzymes produced by fungi. Digestively refractory bark tissue likely was not consumed.

The siderite preserved conifer, *Protocupressinoxylon cupressoides*, from the Middle Jurassic of China, is instructive regarding how reaction tissue can accumulate as layers of cells that encircle the tunnel. The structure of cells lining this tunnel has been interpreted as wound reaction generated by the host plant[43]. However, it is unlikely that nonliving wood could generate live tissue that would produce such a tunnel lining. A more likely possibility is that the tunnel surface was lined by the reconstitution of chewed tissues by the beetle inhabitants. Tunnels in *Protocupressinoxylon* range up to 500 µm in diameter and some contain coprolites of two size classes, one set of 100–165 µm and the other ranging 50–55 µm. The authors suggested that the smaller coprolites were produced by Coleoptera, although Kellogg and Taylor[2] reinterpreted these coprolites as originating from oribatid mites rather than beetles. In the current study, there are more than 10 layers of growth rings that have formed as wound reaction tissue in the specimens YKLP20008a and b (Fig. 1b, c), which indicate that the healing process of the host plants can be extensive, destructive of the terminal tunnel phase, and, in some cases last more than 10 years.

Early Cretaceous petrified wood from the Wealden Formation of Southern England is of additional interest because, like the Jurassic material from China, it shows two types of beetle borings[44]. This size distribution supports the view that multiple inhabitants can occupy tunnel networks, including the possibility that a stressed host tree is liable to be attacked from more than one borer at the same time. Bark beetles have been considered responsible for these borings[45], although it has been argued that the tunnels also could have been produced by larvae belonging to stem group Curculionidae[13,44]. It should be noted that varied, unconventional taxa can be fabricators of borings in fossil

woods. For example, distinctive tunnel-like borings in woods in a freshwater deposit of the Late Cretaceous Wahweap Formation, from the western USA, has been attributed to mayfly nymphs[46].

Supplementary Note 2 | Latest Permian to mid-Mesozoic Wood-Boring Polyphaga

Although the Archostemata has been cited as the culprit clade for fabrication of Permian wood borings, we present evidence that the morphological and ecological evidence points to the Suborder Polyphaga in constructing the highly structured late Permian borings described herein. Two lines of evidence suggest the presence of Polyphaga during the late Permian. First is phylogenetic evidence, including a basal position of the Polyphaga within the Coleoptera[47,48], and second is the probable presence of polyphagan beetles — or at least a beetle morphology very similar to the Polyphaga — are commonly present in several deposits[52–56]. However, within the Polyphaga, family-level fossil data indicate a much more recent clustering of the first appearances of wood-boring beetle lineages during the mid-Mesozoic[47]. Assuming that modern wood-boring life habits were present in early representatives of almost all uniformly wood-boring extant clades, these earliest occurrences of the polyphagan wood-boring habit range from late Middle Jurassic (Callovian Stage, at 165 million-years (MA) in age) for the Buprestidae and Cerambycidae, the Late Jurassic (Oxfordian Stage, ca. 160 Ma) for the Bostrychidae, and earlier Early Cretaceous (Aptian Stage, ca. 120 Ma) for Lymexylidae[13,57,58]. These earliest body-fossil occurrences of major Mesozoic wood-boring beetle lineages indicate host-plant associations of wood-borers were with arborescent gymnosperms. With the possible exception of the last, Aptian, occurrence, arborescent angiosperms were not available as hosts during earlier time intervals[14]. Importantly and based on body-fossil evidence, the earliest, currently known, polyphagan wood-borer lineage with a complex system of a major mother gallery and associated subsidiary larval tunnels are Scolytinae dated at 132.5 Ma in age. This date indicates a ca. 120 Ma gap between the late Permian polyphagan occurrence at ca. 253 Ma and the earliest, currently known, later polyphagan lineage at 132.5 Ma with a

similarly complex wood-boring system of a mother gallery and connected larval tunnels.

Many reports of fossil beetle borings cannot be assigned precisely to any particular living insect clade, especially in the older part of the fossil record. An example is the borings and coprolites contained in the late Permian tree-fern, *Psaronius*, from Yunnan Province, Southwest China[59]. One exception, however, is engravings observed on a tree axis from the Miocene of Shandong Province, East China, that is closely comparable with those made by modern bark beetles[60]. However, the affinity of the host plant is unclear. Notably, an engraving made by a bark beetle, assigned to the genus *Dendroctonus* of the tribe Tomicini, has been recognised in middle Eocene mummified wood of *Larix altoborealis* (Pinaceae) from the Canadian High Arctic[61]. In the latter case, alternative hypotheses could be posed for the associational relationship between particular conifer host and bark beetle lineages. One hypothesis states that a relationship can be traced back to Early Cretaceous representatives of the genus *Larix* (larch), whereas a second hypothesis augurs for a more recent, Paleogene origin. Either scenario would have important implications for the phylogeny of both the plants and their bark-beetle colonisers[27].

Supplementary Note 3 | Full Descriptions of Borings and Their Inclusions

Four wedge-shaped wood pieces were obtained from three tree axes, each containing one or two galleries and associated tunnel networks of beetle borings (Fig. 1a–d and Supplementary Fig. 1a). The borings consist of an expansive mother gallery, which was largely empty of beetle associated frass but contained shelter-seeking fungi and invertebrates that evidently entered after initial beetle occupation. The transverse section of the mother gallery shows a flattened aspect between the secondary xylem (wood) and bark (Fig. 1e). From the mother gallery originated numerous, linear to slightly curvilinear, subsidiary (larval) tunnels that contain frass, including coprolites, and colonising organisms. The subsidiary tunnels are oriented vertically along the axis and are parallel to each other (Fig. 1d). The number of subsidiary tunnels range from six to eleven, each of which typically are ca. 3.6–4.2 mm in diameter. The subsidiary tunnels

often begin from the meristematic cambial tissue layer between the wood and bark. Subsequently, while the larvae are excavating tissues along the cambium layer, their tunnels gradually descend into the wood. Soon thereafter the tunnels are completely embedded within the wood (Fig. 1a–c and Supplementary Fig. 1b–e). Wound-induced reaction tissue produced by the plant host envelops each tunnel series, characterised by a thick response wood that originates from lateral growth of the adjacent cambial tissues for a period lasting ten or more years (Fig. 1b and Supplementary Fig. 1f). Typical fungally induced cell-wall alternations are present around the borings (Fig. 1g). Exquisitely, well-preserved biological inclusions, including fragmentary beetle body remains, mites, coprolites, fungal hyphae and cheliceral elements of miniscule terrestrial chelicerates are commonly observed in these borings (Figs 1h–s and 2; Supplementary Figs 1h and 2–4).

Specimen YKLP20010 has two borings embedded deep within the wood (Fig. 1a and Supplementary Fig. 1a). Two elliptical callus scars corresponding to the wound reactions are visible on the outer surface of the tree axis (Supplementary Fig. 1a, black arrows). This series of borings close to the centre of the axis comprises eleven subsidiary tunnels. The tangential widths of these tunnels ranges from 2.5 to 5 mm (mean = 4.23 mm), each of which gradually enlarges from their beginning toward their end. When the tunnel width is smaller than 5 mm, their outlines assume a tetragonal cross section; when tunnel width exceeds 5 mm, they exhibit a more horizontally flattened, elliptical outline. As the subsidiary tunnels descend into the wood, they are separated from the cambium layer by more than 1.5 mm. The second series of borings identified in this specimen is smaller than the first one, and is completely embedded in wood (Fig. 1a and Supplementary Fig. 1a, white arrow). Thus, the height of the second series of borings is assumed to be slightly less than 55 mm. The two series of borings occur in a displaced temporal succession, reflected by their locations from the axis surface, indicating that the host plant was attacked by beetles at different times during its lifespan. Exoskeletal carapaces of oribatid mites are commonly present in the borings, some of which are covered by silken hyphae of a saprophagous fungus (Fig. 1q, r and Supplementary Fig.

4b–e).

Specimens YKLP20008a (Fig. 1b, e and Supplementary Fig. 1b–e) and YKLP20008b (Fig. 1c) were retrieved from different parts of the same tree axis. The former specimen reveals a series of borings with six subsidiary tunnels that are embedded deep within the wood; the tangential width of the tunnels varies from 3.1 to 3.9 mm (mean = 3.65 mm). Serial thin sections were made at regular intervals that cut transversely through the subsidiary tunnels, eventually reaching the plane of the mother gallery. These transverse sections show that the subsidiary tunnels descend gradually into the wood after developing from the mother gallery (Supplementary Fig. 1b–e). The latter specimen displays a series of borings with seven subsidiary tunnels. The tangential width of these tunnels varies from 3.3 to 4.0 mm (mean = 3.66 mm). Normal centripetal growth rings are present regularly in the host-tree axis before the initiation of the borings (Fig. 1c, white arrows). However, subsequent reaction wood developed after the host-plant tissues were attacked (Fig. 1c, black arrows; and Supplementary Fig. 1g). Wound reaction tissues consist of a large number of parenchyma cells that commonly surround the borings (Supplementary Fig. 1f). Beetle sclerites are commonly recognised in the associated tunnels (Supplementary Fig. 1h, arrow). Specimens of YKLP20008a and YKLP20008b confirm that the same host plant was attacked by beetles at least twice during its lifetime.

Specimen YKLP20009 (Fig. 1d) is a longitudinal section containing nine subsidiary tunnels within the wood that are rectilinear, parallel, and distributed vertically along the host-plant axis. The inner surfaces of the tunnels have a roughened texture and a jagged appearance with plant debris occurring in the tunnel lumen (Fig. 1f). Similar to other specimens, the adjacent cells of the tunnels show typical fungal decay features: (i), the tracheids generally lack detailed microstructure (Fig. 1f); and (ii), prominent cell wall separations of tracheids commonly are recognised (Fig. 1g).

The cambial tissue of the bored region was destroyed completely during tunnel formation. Consequently, the periclinal growth of the tree axis has been suspended in

the wound region of the borings. However, the cambial tissue in other regions continuously produces wood that is directed inwardly, and the bark is oriented outwardly. Due to the asymmetrical division of the cambium layer, the borings are enveloped by lateral growth of the tree axis (Fig. 1c, black arrows). As a consequence, the lateral-most tunnels generally are infilled with xylary tissue that is moulded into the tunnel lumen during wood enrollment (Supplementary Fig. 1g). A substantial volume of parenchyma cells occurs as reaction tissue — a feature that is commonly observed around the borings (Supplementary Fig. 1f).

The above borings contain a variety of biological inclusions that inform interpretation of the ecological relationships between the beetle borers and other organisms within the close confines of a complex tunnel system. Besides fungal hyphal networks (Fig. 1j and Supplementary Fig. 2b–e), exoskeletons of two mite taxa (Fig. 1p, q and Supplementary Fig. 4a–e), sclerites attributable to a chelicerate arthropod (Fig. 1o), and the chorion surfaces of beetle eggs (Fig. 1l and Supplementary Fig. 2p) also were preserved, in addition to the most prominent elements of larval and adult beetle mandibles (Fig. 1h, i and Supplementary Fig. 2a–f). Less conspicuous are somewhat shriveled, often twisted and otherwise altered elements of head, thoracic and abdominal sclerites of the beetle occupant (Supplementary Fig. 2g–o). Other readily identifiable elements include possible body elements and falcate movable digits of chelicerae (Supplementary Fig. 3a–h), and spinose leg fragments (Fig. 1m, n and Supplementary Fig. 3i–o) that cannot be attributed to the beetle borer but presumably originated from other organisms such as mites, pseudoscorpions or other chelicerates seeking shelter in the tunnels (Supplementary Fig. 4f–k). Fungal hyphae and a specimen of a fungivorous mite attributed to the Family Pygmephoridae also were observed (Fig. 1p and Supplementary Fig. 4a). This inventory provides evidence for a nexus of trophic relationships and resource use, such as shelter and diets involving cambial tissue, fungi wood-borers, predators, and other organisms present among a micro-community of organisms inhabiting the axis of a conifer host.

It is notable that fungal hyphae commonly occur in larval tunnels of all examined specimens (Fig. 1s). The fungal hyphae are regularly septate, ca. 150 µm long and 1–2 µm wide, and consist of a main axis attached with numerous short, spine-like branches. The basal part of each hypha is slightly broader, and gradually tapers distally. The fungus prominently shows a growth habit of a mycelial network. Although no reproductive structures were detected, the vegetative morphology of the fungus roughly corresponds to the extant deuteromycetous species *Melanographium spinulosum*[65,66].

Supplementary Note 4 | Locality and Geological Setting

The fossil locality of Shitanjing Coalfield is situated in the northernmost part of the Ningxia Huizu Autonomous Region, Northwest China. Tectonically, this coalfield is part of the western Ordos (also spelled as Erdos or Erduosi) sedimentary basin at the northwestern edge of the North China Block (NCB). The northwestern NCB is characterised by excellent preservation of late Palaeozoic permineralised woods. Although the study of the permineralised wood within this area has a long history[67], only recently have there been intensive studies to understand the taxonomic affinities and anatomical structure of these woods[68–78].

More than a hundred permineralised tree axes from 12 fluvial deposits of early to late Permian age have been collected in the study area. These fossil axes originate from a stratigraphic section encompassing the Shanxi, Lower Shihhotse, Upper Shihhotse and Sunjiagou formations. Several new fossil plant taxa of possible ginkgophyte and coniferophyte affinities have been recognised from this area[68–78]. These plant taxa are strikingly different in taxonomic affinities from those occurring in the palaeocontinents of Angara, Euramerica or Gondwana.

Silica permineralised axes that contain the beetle borings were collected from the middle–upper part of Sunjiagou Formation, of late Permian age. The bed from which the axes were retrieved is equivalent to the Changhsingian Stage, whose duration was ca. 254–252 million-years ago. The Sunjiagou Formation at the Shitanjing locality consists of

a sedimentary succession of fluvial, deltaic and lacustrine environments. The bed yielding the permineralised tree axes is composed of a medium-grained sandstone sequence that is intercalated with mudstone beds. A thin, lenticular limestone bed is present above the wood-bearing bed.

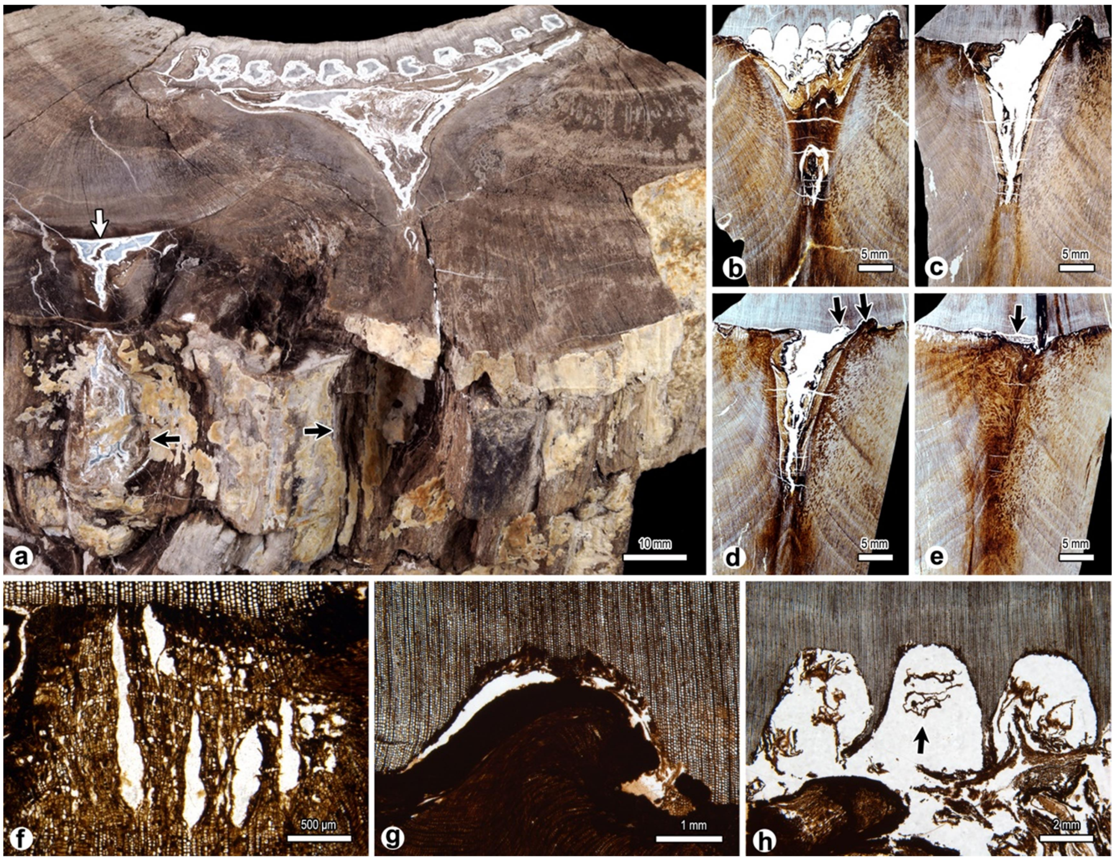

Supplementary Figure 1 | Tunnel networks of late Permian beetle borings from China. (**a**) Slightly oblique view of specimen YKLP20010, showing two networks of borings. At top is a series of eleven subsidiary (larval) tunnels and an associated triangular callus. The white arrow indicates a younger callus with borings embedded within the wood; black arrows indicate two callus enrollments formed by lateral growth of reaction wood. (**b**–**e**) Serial transverse thin sections of specimen YKLP20008a, showing the chronological development of larval tunnels in a series of vertical borings from the top to bottom in a tree axis. Note the presence of six larval tunnels in **b**, four in **c**, two in **d** (arrows), none in **e**, with arrow indicating the mother gallery. (**f**) Parenchyma cells surrounding borings. (**g**) Enrollment of wood infilling a larval tunnel. (**h**) Beetle body sclerites present in the larval tunnels.

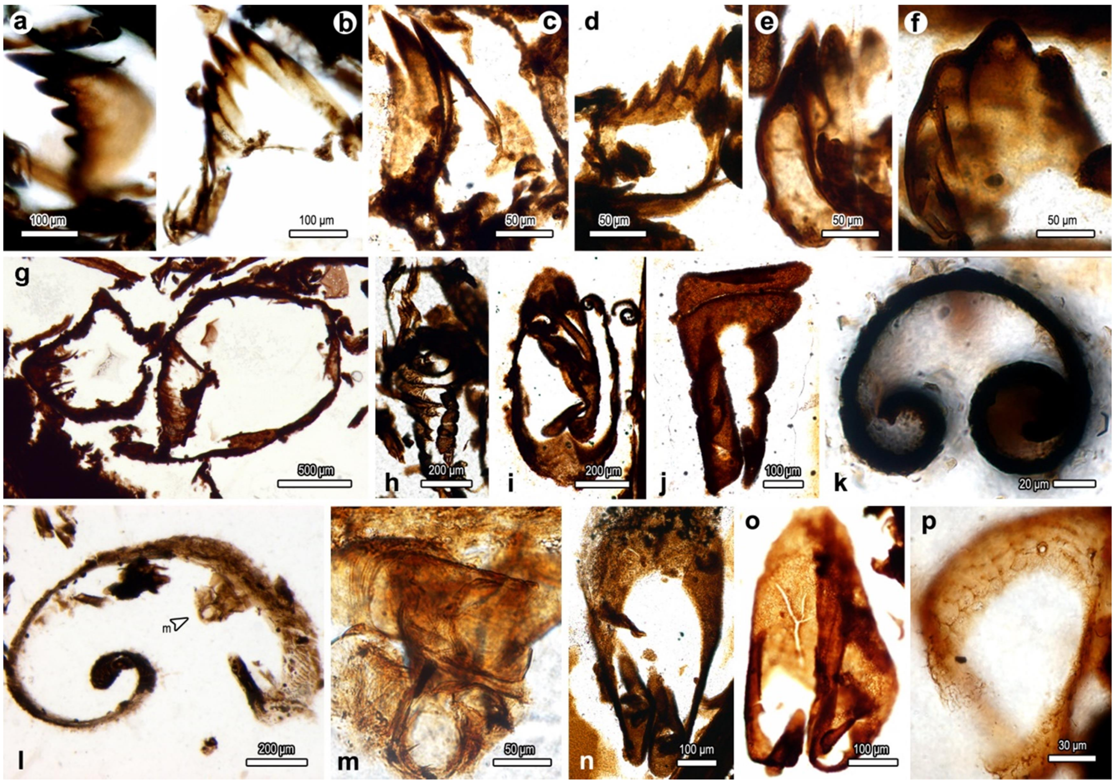

Supplementary Figure 2 | Beetle body fragments in borings from the upper Permian of China. (**a–f**) Multidentate beetle mandibles ranging from fresh falcate incisor form teeth (a b, c) to worn nubbins (d, e, f). (**g**–**o**) Variously sectioned body sclerites. (**p**) The chorion layer of a beetle.

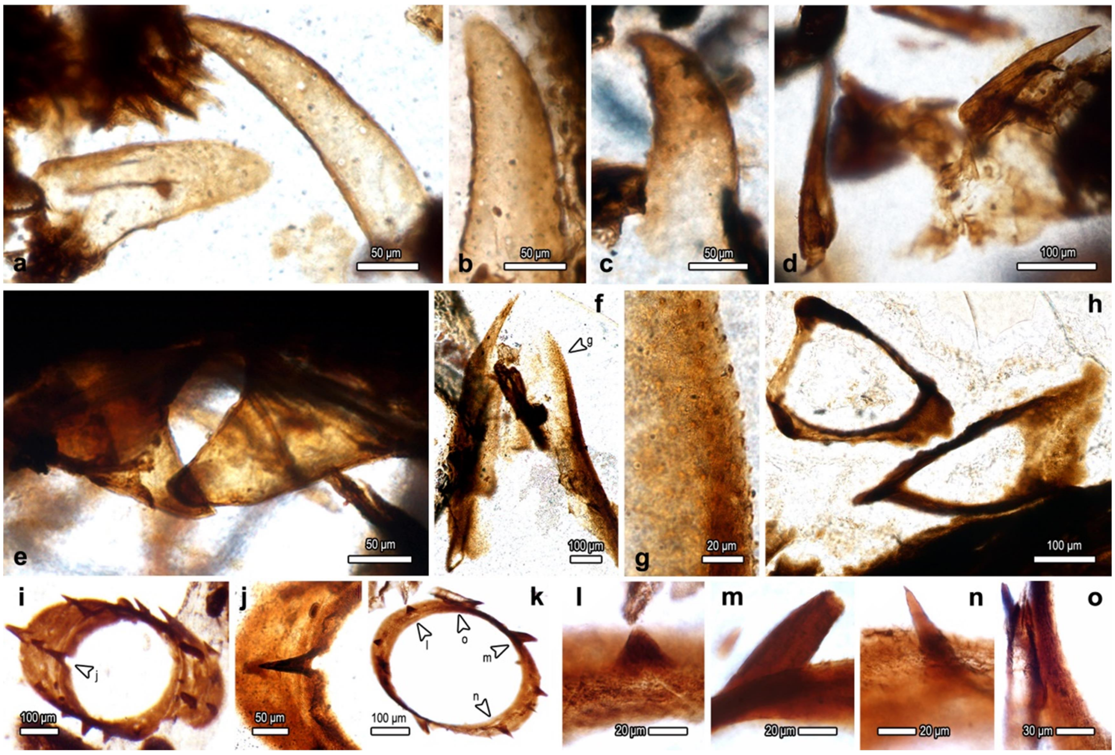

Supplementary Figure 3 | Arthropod inclusions in beetle borings from the upper Permian of China. (**a**–**d**) Single and probably paired cheliceral elements. (**e**–**h**) Falcate cheliceral elements, representing the movable digit, shown possibly paired (e), and in lateral (f, g) and cross (h) sections. (**i**–**o**) Thoracic leg elements showing simple spines. These elements are attributable to pseudoscorpions, harvestmen or mites[S62].

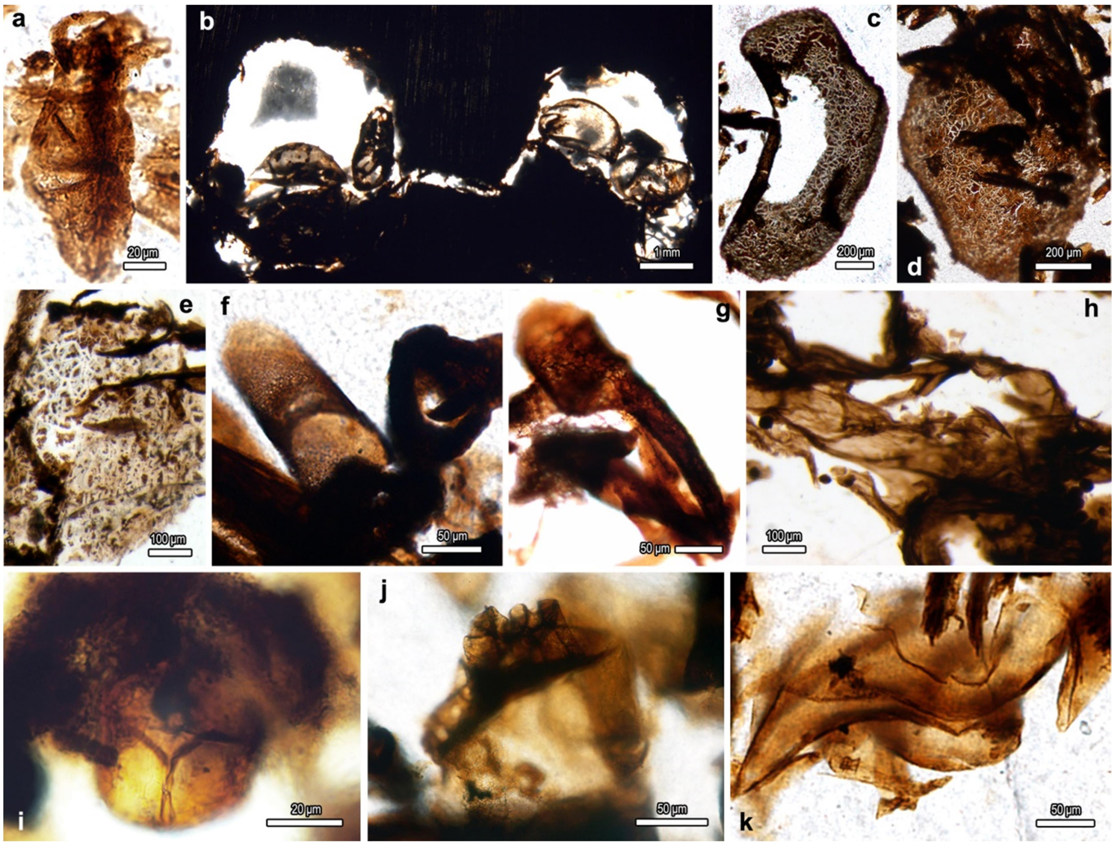

Supplementary Figure 4 | Biological inclusions in beetle borings from the upper Permian of China. (**a**) A mite likely affiliated with the Pygmephoridae[63], modern representatives which are mycetophagous and associated with wood boring beetles[64]. (**b**) Four carapaces of large oribatid mites in adjacent larval tunnels. (**c**–**e**) Degraded carapaces of oribatid mites enveloped by hyphal networks of saprophagous fungi. (**f**–**k**) unidentifiable body elements.

SUPPLEMENTARY REFERENCES

1. Solomon, J. D. *Guide to Insect Borers in North American Broadleaf Trees and Shrubs.* Forest Service Agriculture Handbook AH-706 (Washington, 1995).
2. Kellogg, D. W. & Taylor, E. L. Evidence of oribatid mite detritivory in Antarctica during the late Paleozoic and Mesozoic. *J. Paleontol.* **78,** 1146–1153 (2004).
3. Johnson, W. T. & Lyon, H. H. *Insects that Feed on Trees and Shrubs*. Second edn. (Cornell Univ. Press, 1991).
4. Vilhelmsen, L. & Turrisi, G. F. Per arborem ad astra: morphological adaptations to exploiting the woody habitat in the early evolution of Hymenoptera. *Arthro. Struct. Dev.* **40,** 2–20 (2011).
5. Labandeira, C. C., Phillips, T. L. & Norton, R. A. Oribatid mites and the decomposition of plant tissues in Paleozoic coal-swamp forests. *Palaios* **12,** 319–353 (1997).
6. Labandeira, C. C. & Phillips, T. L. Stem borings and petiole galls from Pennsylvanian tree ferns of Illinois, USA: implications for the origin of the borer and galling functional-feeding-groups and holometabolous insects. *Palaeontographica (A)* **264,** 1–84 (2002).
7. Arnold, C. A. Bark structure of *Callixylon*. *Bot. Gaz.* **90,** 427–431 (1930).
8. Scheckler, S. E., Meyer-Berthaud, B. & Galtier, J. Secondary phloem of the Late Devonian progymnosperm tree *Archaeopteris*. *Abstracts, Plants and People* **2001,** 69. (2001).
9. Feng, Z., Schneider, J. W., Labandeira, C. C., Kretzschmar, R. & Rößler, R. A specialized feeding habit of early Permian oribatid mites. *Palaeogeogr., Palaeoclimatol., Palaeoecol.* **417,** 212–215 (2015).
10. Wan, M., Yang, W., Liu, L. J. & Wang, J. Plant–arthropod and plant–fungus interactions in late Permian gymnospermous woods from the Bogda Mountains, Xinjiang, northwestern China. *Rev. Palaeobot. Palynol.* **235,** 120–128 (2016).
11. Rößler, R. & Fiedler, G. Fraßspuren an permischen Gymnospermen–Kieselhölzern–Lebenszeichen von Arthropoden im Oberrotliegend von Chemnitz. *Veröff. Mus. Naturk. Chemnitz* **19,** 27–34 (1996).

12. Scott, A. C., Stephenson, J. & Paterson, S. Evidence of pteridophyte–arthropod interactions in the fossil record. *Proc. R. Soc. Edinburgh (B)* **86,** 133–140 (1985).
13. Chaloner, W. G., Scott, A. C. & Stephenson, J. Fossil evidence for plant–arthropod interactions in the Palaeozoic and Mesozoic. *Phil. Trans. R. Soc. London (B)* **333,** 177–186 (1991).
14. Labandeira, C. C. The four phases of plant–arthropod associations in deep time. *Geol. Acta* **4,** 409–438 (2006).
15. Labandeira, C. C. Silurian to Triassic plant and insect clades and their associations: new data, a review, and interpretations. *Arthro. Syst. Phylo.* **64,** 53–94 (2006).
16. Dunn, M. T., Rothwell, G. W. & Mapes, G. On Paleozoic plants from marine strata: *Trivenia arkansana* (Lyginopteridaceae) gen. et sp. nov., a lyginopterid from the Fayetteville Formation (middle Chesterian/Upper Mississippian) of Arkansas, USA. *Am. J. Bot.* **90,** 1239–1252 (2003).
17. Scott, A. C. & Taylor, T. N. Plant/animal interactions during the Upper Carboniferous. *Bot. Rev.* **49,** 259–307 (1983).
18. Taylor, E. L. Secondary phloem anatomy in cordaitean axes. *Am. J. Bot.* **75,** 1655–1666 (1988).
19. Maslen, A. J. The structure of *Mesoxylon sutcliffii* Scott. *Ann. Bot.* **25,** 381–412 (1911).
20. Kukalová-Peck, J. & Beutel, R. G. Is the Carboniferous †*Adiphlebia lacoana* really the "oldest beetle"? Critical reassessment and description of a new Permian beetle family. *Eur. J. Entomol.* **109,** 633–645 (2012).
21. Kirejtshuk, A. G., Poschmann, M., Prokop, J., Garrouste, R. & Nel, A. Evolution of the elytral venation and structural adaptations in the oldest Palaeozoic beetles (Insecta: Coleoptera: Tshekardocoleidae). *J. Syst. Palaeontol.* **12,** 575–600 (2014).
22. Toussaint, E. F. A., Seidel, M., Arriaga-Varela, E., Hajek, J., Kral, D., Sekerka, L., Short, A. E. Z. & Fikacek, M. The peril of dating beetles. *Syst. Entomol.* **42,** 1–10 (2017).
23. Béthoux, O. The oldest beetle identified. *J. Paleontol.* **83,** 931–937 (2009).
24. Naugolnykh, S. V. & Ponomarenko, A. G. Possible traces of feeding by beetles in coniferophyte wood from the Kazanian of the Kama River Basin. *Paleontol. J.* **44,** 468–

474 (2010).

25. Zavada, M. S. & Mentis, M. T. Plant–animal interaction: the effect of Permian megaherbivores on the glossopterid flora. *Am. Midl. Nat.* **127,** 1–12 (1992).

26. Weaver, L., McLoughlin, S. & Drinnan, A. N. Fossil woods from the Upper Permian Bainmedart Coal Measures, northern Prince Charles Mountains, East Antarctica. *J. Austral. Geol. Geophys.* **16,** 655–676 (1997).

27. Taylor, T. N., Taylor, E. L. & Krings, M. *Paleobotany: The Biology and Evolution of Fossil Plants*. Second edn. (Academic Press, 2009).

28. Linck, O. Fossile Bohrgänge (*Anobichnium simile* n. g., n. sp.) an einem Keuperholz. *Neues. Jb Mineral. Paläontol. Monat.* **1949,** 180–185 (1949).

29. Ash, S. R. Evidence of arthropod–interactions in the Upper Triassic of the southwestern United States. *Lethaia* **28,** 237–248 (1997).

30. Walker, M. V. Evidence of Triassic insects in the Petrified Forest National Monument, Arizona. *Proc. US Natl. Mus.* **85,** 137–141 (1938).

31. Ash, S. R. & Savidge, R. A. The bark of the Late Triassic *Araucarioxylon arizonicum* tree from Petrified Forest National Park, Arizona. *IAWA J.* **25,** 349–368 (2004).

32. Tapanila, L. & Roberts, E. M. The earliest evidence of holometabolan insect pupation in conifer wood. *PLoS ONE* **7,** e31668 (2012).

33. Hasiotis, S. & Dubiel, R. F. Continental trace fossils of the Upper Triassic Chinle Formation, Petrified Forest National Park, Arizona. *New Mexico Mus. Nat. Hist. Sci. Bull.* **3,** 175–178 (1993).

34. Hasiotis, S. T., Dubiel, R. F., Kay, P. T., Demko, T. M., Kowalska, K. & McDaniel, D. Research update on hymenopteran nests and cocoons, Upper Triassic Chinle Formation, Petrified Forest National Park, Arizona. *Natl. Park Ser. Paleontol. Res. Tech. Rept.* **NPS/NRGRD/GRDTR-98/01** (1998).

35. Hasiotis, S. F. Complex ichnofossils of solitary and social soil organisms: understanding their evolution and roles in terrestrial paleoecosystems. *Palaeogeogr., Palaeoclimatol., Palaeoecol.* **192,** 259–320 (2003).

36. Lucas, S. G., Minter, N. J. & Hunt, A. P. Re-evaluation of alleged bees' nests from the Upper Triassic of Arizona. *Palaeogeogr., Palaeoclimatol., Palaeoecol.* **286,** 194–201

(2010).

37. Haack, R. & Slansky, F. Nutritional ecology of wood-feeding Coleoptera, Lepidoptera, and Hymenoptera. in *Nutritional Ecology of Insects, Mites, Spiders, and Related Invertebrates.* (ed. Slansky, F.) 449–486 (Wiley & Sons, 1987).

38. Gullan, P. J. & Cranston, P. S. *The Insects: An Outline of Entomology*. 5th edn. (Wiley-Blackwell, 2014).

39. Hamilton, W. Evolution and diversity under bark. *Symp. Roy. Entomol. Soc. London* **9,** 154–175 (1978).

40. Fukuda, A. Biology of *Tenomerga mucida*. *Trans. Nat. Hist. Soc. Formosa* **31,** 394–399 (1941).

41. Ross, D. A. & Pothecary, D. D. Notes on adults, eggs and first-instar larvae of *Priacma serrata* (Coleoptera: Cupedidae). *Canad. Entomol.* **102,** 346–348 (1970).

42. Fukuda, A. On the larva of *Cupes clathratus*. *Trans. Natl. Hist. Soc. Formosa* **29,** 75–82 (1939).

43. Zhou, Z. Y. & Zhang, B. L. A sideritic *Protocupressinoxylon* with insect borings and frass from the Middle Jurassic, Henan, China. *Rev. Palaeobot. Palynol.* **59,** 133–143 (1989).

44. Jarzembowski, E. A. A boring beetle from the Wealden of the Weald. in *Evolutionary Paleobiology of Behaviour and Coevolution.* (ed. Boucot, A.) 373–376 (Elsevier, 1990).

45. Crowson, R. A. *The Biology of the Coleoptera.* (Academic Press, 1981).

46. Moran, K., Hilbert-Wolf, H. L., Golder, K., Malenda, H. F., Smith, C. J., Storm, L. P., Simpson, E. L., Wizevich, M. C. & Tindall, S. E. Attributes of the wood-boring trace fossil *Asthenopodichnium* in the Late Cretaceous Wahweap Formation, Utah, USA. *Palaeogeogr., Palaeoclimatol., Palaeoecol.* **297,** 662–669 (2010).

47. Lawrence, J. F., Ślipiński, A., Seago, A. E., Thayer, M. K., Newton, A. F. & Marvaldi, A. E. Phylogeny of the Coleoptera based on morphological characters of adults and larvae. *Ann. Zool.* **61,** 1–217 (2011).

48. Hunt, T., Bergsten, J., Levanicova, Z., Papadopoulou, A., St. John, O., Wild, R., Hammond, P. M., Ahrens, D., Balke, M., Caterino, M. S., Gómez-Zurita, J., Ribera, I., Barraclough, T. G., Bocakova, M., Bocak, L. & Vogler, A. P. A comprehensive phylogeny

of beetles reveals the evolutionary origins of a super radiation. *Science* **318,** 1913–1916 (2007).

49. Ponomarenko, A. G. The first beetles (Permosynidae, Coleoptera) from the Upper Tatarian of European Russia. *Paleontol. J.* **37,** 65–68 (2003).

50. Fedorenko, D. N. & Ponomarenko, A. G. First records of hindwings of Permian beetles (Coleoptera). *Paleontol. J.* **46,** 164–170 (2012).

51. Ponomarenko, A. G. New beetles (Insecta, Coleoptera) from Vyazniki locality, terminal Permian of European Russia. *Paleontol. J.* **45,** 414–422 (2011).

52. Ponomarenko, A. G. New Triassic beetles (Coleoptera) from northern European Russia. *Paleontol. J.* **42,** 600–606 (2008).

53. Ponomarenko, A. G. Beetles (Insecta, Coleoptera) of the Late Permian and Early Triassic. *Paleontol. J.* **38,** S185–S196 (2004).

54. Chatzimanolis, S., Grimaldi, D. A., Engel, M. S. & Fraser, N. C. *Leehermania prorova*, the earliest staphyliniform beetle, from the Late Triassic of Virginia (Coleoptera: Staphylinidae). *Am. Mus. Novit.* **3761,** 1–28 (2012).

55. Meller, B., Ponomarenko, A. G., Vasilenko, D. V., Fischer, T. C. & Aschauer, B. First beetle elytra, abdomen (Coleoptera) and a mine trace from Lunz (Carnian, Late Triassic, Lunz-am-See, Austria) and their taphonomical and evolutionary aspects. *Palaeontology* **54,** 97–110 (2011).

56. Ponomarenko, A. G. Ecological evolution of beetles (Insecta: Coleoptera*). Acta Zool. Cracovien.* **46(Suppl. Fossil Insects),** 319–328 (2003).

57. Labandeira, C. C. Why did terrestrial insect diversity not increase during the angiosperm radiation? Mid-Mesozoic, plant-associated insect lineages harbor clues. in *Evolutionary Biology: Genome Evolution, Speciation, Coevolution and Origin of Life.* (ed. Pontarotti, P.) 261–299 (Springer, 2014).

58. Legalov, A. A. Checklist of Mesozoic Curculionoidea with description of new taxa. *Balt. J. Coleopt.* **10,** 71–101 (2010).

59. D'Rozario, A., Labandeira, C. C., Guo, W. Y., Yao, Y. F. & Li, C. S. Spatiotemporal extension of the Euramerican *Psaronius* component community to the Late Permian of Cathaysia: In situ coprolites in a *P. housuoensis* stem from Yunnan Province,

southwest China. *Palaeogeogr., Palaeoclimatol., Palaeoecol.* **306,** 127–133 (2012).

60. Guo, S. A Miocene trace fossil of insect from Shanwang Formation in Linqu, Shandong. *Acta Palaeontol. Sin.* **31,** 739–742 (1991).

61. Labandeira, C. C., LePage, B. A. & Johnson, A. H. A *Dendroctonus* bark engraving (Coleoptera: Scolytidae) from a Middle Eocene *Larix* (Coniferales: Pinaceae): early or delayed colonization? *Am. J. Bot.* **88,** 2026–2039 (2001).

62. Eisenbeis, G. & Wichard, W. *Atlas on the Biology of Soil Arthropods.* (Springer-Verlag, 1987).

S63. Rahiminejad, V., Hajiqanbar, H. & Fathipour, Y. Two new species of the genus *Elattoma* (Acari: Heterostigmatina: Pygmephoridae) phoretic on *Morimus verecundus* (Coleoptera: Cerambycidae) from Iran. *Zootaxa* **2903,** 48–56 (2011).

64. Hofstetter, R. W., Dinkins-Bookwalter, J., Davis, T. S. & Klepzig, K. D. Symbiotic associations of bark beetles. in *Bark Beetles: Biology and Ecology of Native and Invasive Species.* (eds Vega, F. E. & Hofstetter, R. W.) 209–245 (Elsevier, 2015).

65. Hughes, S. J. Revisiones Hyphomycetum aliquot cum appendice de nominibus rejiciendis. *Can. J. Bot.* **36,** 727–836 (1958).

66. Morris, E. F. The synnematous genera of the Fungi Imperfecti. Series in the Biological Sciences, no. 3 (Western Illinois Uni. Press, 1963).

67. Sze, H. C. A fossil wood from Ningxia. *Bull. Geol. Soc. China* **26,** 101–104 (1946).

68. Yang, J. Y., Shen, J. J., Chen, Y. X., Wei, H. B., Kerp, H. & Feng, Z. The bark anatomy of *Ningxiaites specialis* from the Permian of China. *Rev. Palaeobot. Palynol.* **240,** 11–21 (2017).

69. Wei, H. B., Feng, Z., Yang, J. Y., Chen, Y. X. & He, X. Y. Specialised emission pattern of leaf traces in a late Permian (253 million-years old) conifer. *Sci. Rep.-UK* **5,** 12405 (2015).

70. Feng, Z., Wang, J., Rößler, R., Kerp, H. & Wei, H. B. Complete tylosis formation in a latest Permian conifer stem. *Ann. Bot.* **111,** 1075–1081 (2013).

71. Feng, Z., Wang, J., Liu, L. J. & Rößler, R. A novel coniferous tree trunk with septate pith from the Guadalupian (Permian) of China: ecological and evolutionary significance. *Int. J. Plant Sci.* **173,** 835–848 (2012).

72. Feng, Z. *Ningxiaites specialis*, a new woody gymnosperm from the uppermost Permian of China. *Rev. Palaeobot. Palynol.* **181,** 34–46 (2012).

73. Feng, Z., Wang, J. & Rößler, R. A unique gymnosperm from the latest Permian of China, and its ecophysiological implications. *Rev. Palaeobot. Palynol.* **165,** 27–40 (2011).

74. Feng, Z., Wang, J. & Rößler, R. *Palaeoginkgoxylon zhoui*, a new ginkgophyte wood from the Guadalupian (Permian) of China and its evolutionary implications. *Rev. Palaeobot. Palynol.* **162,** 146–158 (2010).

75. Feng, Z., Wang, J. & Liu, L. J. First report of oribatid mite (arthropod) borings and coprolites in Permian woods from the Helan Mountains of northern China. *Palaeogeogr., Palaeoclimatol., Palaeoecol.* **288,** 54–61 (2010).

76. Feng, Z., Wang, J. & Shen, G. L. *Zalesskioxylon xiaheyanense* sp. nov., a gymnospermous wood of the Stephanian (Late Carboniferous) from Ningxia, northwestern China. *J. Asian Earth Sci.* **33,** 219–228 (2008).

77. Wang, J. Permian wood from Inner Mongolia, north China: with special reference to Palaeozoic climate change of North China Block. *Palaeobotanist* **49,** 353–370 (2000).

78. Wang, S. J., Hu, Y. F. & Cui, J. Z. A new species of *Araucarioxylon* Kraus from the early Early Permian, Nei Mongol, China. *Acta Bot. Sin.* **42,** 427–432 (2000).
